# Cytomegalovirus Infections among African-Americans

**DOI:** 10.1186/1471-2334-8-107

**Published:** 2008-08-01

**Authors:** Isca R Wilms, Al M Best, Stuart P Adler

**Affiliations:** 1 Department of Pediatrics, Virginia Commonwealth University, Richmond, Virginia, USA; 2 Department of Biostatistics, Virginia Commonwealth University, Richmond, Virginia, USA

## Abstract

**Background:**

Since African-Americans have twice the prevalence of cytomegalovirus (CMV) infections as age-matched Caucasians we sought to determine the ages and possible sources of infection of African-American children.

**Methods:**

Subjects were 157 African-American healthy children and adolescents and their 113 household adults in Richmond VA. Families completed a questionnaire, provided saliva for antibody testing, and adolescents were interviewed regarding sexual activity.

**Results:**

Regardless of age CMV seropositivity was not associated with gender, breast feeding, health insurance, sexual activity, or household income, education, or size. In the final regression model, prior CMV infection in adults was over two-fold higher than in children (chi-square = 18.8, p < 0.0001). At one year of age the CMV seropositivity rate was 11% (95%CI = 4% – 24%) and increased 1.8% each year until age 13 years. Between ages 13 and 20 years the CMV seropositivity rate remained between 22% and 33%. For adults, the CMV seropositivity rate was 84% in 21 year olds (95%CI = 69%–.92%). There was no association between CMV infections of the children and their mothers but CMV infections among siblings were associated.

**Conclusion:**

We observed that African-American children had CMV seroprevalence rates by age 20 years at less than one-half of that of their adult mothers and caregivers. Sibling-to-sibling transmission was a likely source of CMV infections for the children. The next generation of African-American women may be highly susceptible to a primary CMV infection during pregnancy and may benefit from a CMV vaccine.

## Background

Studies completed in the 1980s and early 1990s all found that in the United States African-American adults have twice the prevalence of CMV infections than have age-matched Caucasians [1-7]. In these studies African-Americans had seropositivity rate of between 75% and 100% [7]. In Richmond VA in the 1980s we also observed a higher rate of CMV infections among the African-American population as compared to Caucasians [1]. Two studies performed at different times reported the prevalence of CMV infections between 1986 and 1994 among African-Americans between ages 6 and 22 years [2,4]. The rate of CMV seropositivity increased with age and ranged from 30 to 60%. These rates were also nearly twice the rate for age-matched Caucasians. The reasons for the increased rate of CMV infections among African-Americans as compared to Caucasians are unknown. Therefore we performed a study among African-American-American children and adolescents to determine the possible sources of infection.

## Methods

### Study population

Subjects were African-American 157 children and adolescents (between 5 months and 20 years of age) and their 113 family members who were recruited from 121 families at one private pediatric practice (106 families) and one public pediatric practice (15 families), each of which served inner city families in Richmond VA. The average family size was 4.28 members (range 1 to 6). Baseline demographics of subjects recruited from each practice type were similar. Recruitment occurred from November, 2005 until July, 2006. Only 4 subject families declined to participate. The index child was the one seeking medical consultation. When two or more children were present, the oldest child was the index child. The parent or guardian of each index child provided written informed consent for CMV testing and the completion of a demographic questionnaire. All of the household members and siblings present were tested for seropositivity. The questionnaire included: household size, income level, whether the family and/or child had health insurance, breastfeeding for the children, and the highest level of education within the immediate household. Index subjects greater than 11 years of age were interviewed privately by the attending physician and a second time by one of the investigators (I.W.) about sexual activity, birth control, and condom use. US 2000 census data were obtained from the US Census Bureau, Department of Commerce, Washington, DC.

### Laboratory assays

Serologic status to CMV was determined using saliva. At least 100 uL of saliva was collected in 15 mL conical tubes by either expectoration, or for those unable to expectorate, via bulb suction syringe. Saliva was stored at -20°C. The saliva samples were assayed by enzyme immunoassay for IgG to CMV gB as previously described [8,9]. IgG antibodies to gB move passively from serum to saliva and are present in direct proportion to their concentration in serum [8]. The salivary assay detects IgG to gB when serum neutralizing titers to CMV are ≥ 1:64 in the serum. Serum neutralizing titers in seropositive sera are ≥ 1:256. To avoid measuring maternal antibodies enrolled children were greater than 5 months of age. Passively acquired maternal antibody has a half life of approximately 28 days thus by 5 months of age only 3% of maternal antibody levels are present in an infant serum. This level would be too low to detect in saliva [8].

### Statistical analysis

Subjects are referred to as adults if they were either one of the caregivers (four caregivers were between 19 and 21 years of age) in the family or were 21 years of age or older. Subjects were referred to as children if they were less than 21 years of age (two children were 19 and 20 years of age). The presence of antibodies to CMV (seropositivity) was modeled using repeated-measures logistic regression (GENMOD procedure in SAS version 9.1.3 SP3) with an exchangeable correlation structure to account for within-family correlation. The final model included the following terms: a linear and quadratic trend for the age of the children, a linear trend for the age of the adults, with a different intercept for children and adults. Likelihood-ratio chi-square tests were used to test for significance.

This study was approved by the Virginia Commonwealth University Institutional Review Board. Informed written consent or assent was obtained from all subjects and studies were conducted in accordance with human experimentation guidelines of the US Department of Health and Human Services.

## Results

**Figure 1:**
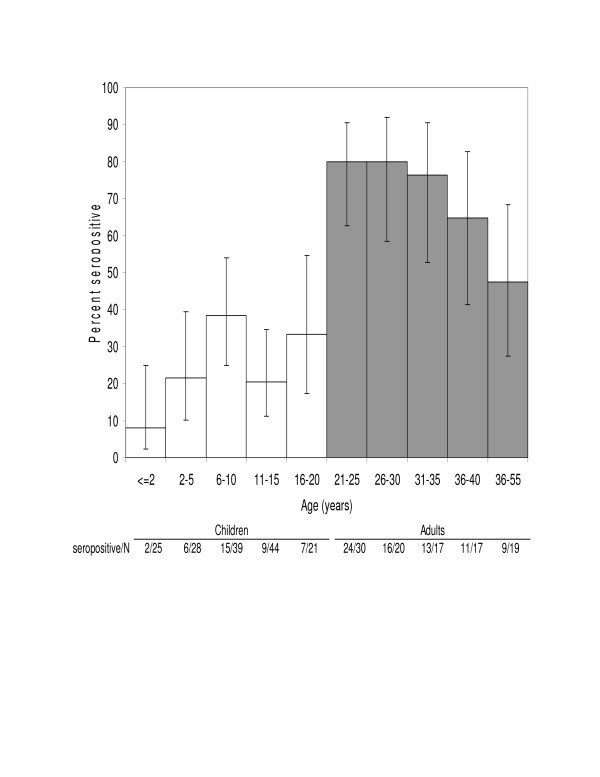
Relationship between age and the rate of CMV seropositivity for 103 adults and 157 children. 95% confidence intervals.

The characteristics of the study population are listed in Table 1. CMV seropositivity and age were determined for 157 children in 121 families. The average age of the children was 8.37 years (standard deviation = ± 5.33 years). For all subjects a multiple logistic regression analysis was used to determine the p-value for the association between each subject characteristic listed in table 1 and age and CMV seropositivity. For children, the only characteristic associated with CMV seropositivity was age (chi-square = 5.76, df = 2, p = 0.056), with both the linear (p = 0.023) and quadratic trends (p = 0.042) both significant (Figure 1).

**Table 1:** Characteristics of the Study Subjects by CMV seropositivity.

	No. of Children (N = 157 CMV status^+^					No. of Adults (N = 113) CMV status^+^				
Characteristic	Pos.	Neg.	Total	% Pos.	p-value	Pos.	Neg.	Total	% Pos.	p-value

Gender					0.54					098
Male	23	58	81	28		73	32	105	70	
Female	16	60	76	21		5	3	8	63	

Breast fed					0.54					
No	16	68	84	19						
Yes	16	32	48	33						

Yearly family income					0.66					0.59
Under $20,000	14	40	54	26		24	14	38	63	
$20,000 to < $40,000	9	21	30	30		16	4	20	80	
$40,000 to < $60,000	6	14	20	30		10	7	17	59	
$60,000 to < $80,000	1	5	6	17		3	2	5	60	
$80,000 or more	2	2	4	50		2	1	3	67	

Health insurance					0.89					0.41
No	2	6	8	25		5	3	8	63	
Yes	31	80	111	28		54	26	80	68	

Family education					0.96					0.41
Completed 9^th ^or less	1	3	4	25		2	2	4	33	
Completed 10^th ^or 11^th^	4	7	11	36		4	4	8	50	
Graduated High School	14	30	44	32		23	8	31	74	
Post high school work	10	36	46	22		23	11	34	68	
Graduated college	4	12	16	25		9	4	13	69	

Household size					0.20					0.89
2	13	44	57	23		28	17	45	62	
3	12	47	59	20		27	11	38	71	
4	6	16	22	27		16	1	17	94	
5+	8	11	19	42		7	6	13	54	

* p-values are reported from repeated-measures logistic regression to account for within family correlation. All p-values test for the significance of the characteristic after adjusting for age, in both children and adults, and the quadratic trend in age for children.

^+ ^Pos = CMV seropositive. Neg. = CMV seronegative. %Pos = the percentage of all test results that were positive.

For adults age was also associated with the CMV seropositivity rate (Figure 1). The average age of the 103 adults who reported their age was 33 years (range 19 to 53 years, SD = 8.75). Age was linearly related to CMV seropositivity (p = 0.027) but no quadratic trend was identified (p = 0.30). After age was taken into account no other characteristics were related to CMV seropositivity.

Of 62 adolescents, only 6 reported being sexually active. Sexual activity was unrelated to CMV seropositivity (chi-square = 0.05, p = 0.82). Of the six sexually active adolescents each had only a single partner and condoms were used.

For the index subjects there were 105 female (104 mothers and 1 aunt) and 8 male caregivers. There was no evidence for a relationship between the CMV seropositivity rate of the index subject and the mother or the female caregiver (data not shown) or between the seropositivity rate of all the children in each household and the mother or caregiver (Table 2).

**Table 2:** The Association of CMV Status between 104 Mothers and their Children.

	Mothers			
Household children	CMV positive	CMV negative	Total tested	Per cent positive
Number CMV positive	30	24	54	55*
Number CMV negative	50	36	86	58

*Not significantly different from the rate of seronegative children, Fisher's exact p-value = 0.86

For families with 2 or more children living at home, CMV seroprevalence among siblings was associated (Table 3). This was independent of the number or ages of the children in the household. This within-family dependence was taken into account in all of the primary analyses.

**Table 3:** The Association of CMV Status between Index Children and their Siblings^+^.

	Index children			
Household siblings	Number CMV positive	Number CMV negative	Total tested	Per cent positive
Number CMV positive	8	2	10	80*
Number CMV negative	1	26	27	3

^+^From 30 families.

*Significantly different from the rate of seronegative siblings, Fisher's exact p-value < 0.0001.

In the final regression model, CMV seroprevalence of adults was higher than children (chi-square = 18.8, p < 0.0001) and for children CMV seropositivity increased with age (Figure 1). At age one year the CMV seropositivity rate was 11% (95%CI = 4% – 24%) and it increased approximately 1.8% each year until approximately age 13 years. Between ages 13 years and 20 years the CMV seropositivity rate remained between 22% and 33% (Table 2 and Figure 1). For younger adults, the CMV seropositivity rate was approximately 84% (95%CI = 69%–92%) in 21 year olds (Figure 1).

Finally, we compared the data from the 2000 US census for all African-Americans residing in Richmond, VA, the state of Virginia and the US to our subject group. The groups were similar for gender distribution, average household size, education levels, and income (Table 4).

**Table 4:** Comparison of Study Subjects to African-Americans Locally, State-wide, and Nationally.

	Group			
Characteristic	Study subjects	City of Richmond *	Virginia (entire state)*	US population*

Average number of persons/household	2.9	2.5	2.7	3.0
Median household income	$29,000	$25,292	$32,080	$29,423
Per cent of female children	51.6	51.5	49.8	49.3

Percent of Household adults by education level				

< 9^th ^grade	1	5	5	8
9^th ^to 12^th ^grade	13	24	19	12
High school or equivalency	39	34	32	29
Some college or associate degree	34	27	30	27
Bachelor's or postgraduate degree	13	10	14	24

*From the 2000 US Census.

## Discussion

Two novel observations emerge from this study. The first is that the cohort of the current generation of African-American children and adolescents in Richmond VA has lower rates of CMV seroprevalence than the older generation comprising their parents and caretakers. We observed a rate of CMV infections for female adults and caregivers that averaged 70%. This rate of seropositivity is similar to those previously reported by us for Richmond and is similar to those reported for adults in other US cities [1-6]. Further, in a population-based survey of African-Americans for the entire US, the average rate of seropositivity for African-American adults was 75.8% [4]. All of the published adult data were obtained in the late 1980s and early 1990s. A study conducted in the late 1980s in Houston reported infection rates for African-Americans between 6 and 22 years of age which increased with age from 30% to 75% [2]. Similarly, in the US population-based study, infection rates for African-Americans tested between 1988 and 1994 from 6 to 20 years of age ranged from 40% to 65% [4]. These rates are similar to those we observed for the current generation of adult females.

Ours is the first recent study to determine CMV seroprevalence rates for the current generation of lower socioeconomic African American children and adolescents. We did not measure the seroprevalence rates in age-matched Caucasians. However, the low rates we observed for lower socioeconomic African American children and adolescents were nearly identical to the low seroprevalence rates reported for Caucasians children and adolescents measured for the entire US population between 1988 and 1994 and for Caucasian children measured in Houston Texas in the late 1980s [2,4]. In contrast, in these studies the seroprevalence rates for African-American children were significantly higher than age-matched Caucasians. Thus our data suggest the current generation of African-American and Caucasian children will have similar CMV seroprevalence rates.

Our observations may be explained by two factors. First, our subject sample may not be representative of the population of African-Americans residing in Richmond, VA or elsewhere especially since we selected subjects seeking medical consultation. This seems unlikely for two reasons. As discussed above, we observed seroprevalence rates for the adults who were predominantly female which were similar to those previously reported for Richmond and other US cities [1-6]. Second, our population was similar in demographic characteristics to that described by the US census for African-Americans in residing the City of Richmond, the state of Virginia, and the nation.

A second more likely reason we observed a lower rate of seroprevalence among African-American children may relate to the infrequent sexual activity reported by our adolescents. The preadolescent seroprevalence rates we observed were similar to those reported in the Houston study (30%–40%) and in the population-based US study (40%) [2-4]. However, in both these studies there was a marked increase in infections rates among adolescents. Sexual activity during adolescence has been frequently associated with increased rates of CMV infection [6,7,10]. Hence our results suggest a decrease in CMV infection in the current generation of African-Americans may have occurred due to decreased sexual activity, increased condom use, or a reduced number of sexual partners during adolescence. AIDS awareness may in part be responsible for this and it will be important to determine if similar changes in sexual behavior and CMV infection rates is also occurring among lower socioeconomic African-American children and adolescents in other cities.

Other factors such the number of children per household and/or changes in hygienic practices may also account for the reduced rate of CMV seroprevalence infections we observed in the current generation of African-American adolescents and children as compared to the higher rates observed in their parents and in older studies.

We observed a decline in the seropositivity rate with age for African-American adults between 40 and 45 years of age. The significance, if any, of this observation is uncertain This decline was not due to reduced antibody levels in saliva associated with aging since antibody titers to CMV persist for life and actually increased throughout adulthood with highest levels in the elderly [11]. Although this decline may represent another cohort of older African-Americans with lower infection rates, the number of subjects in this age range was low and thus the 95% confidence intervals were wide and as shown in figure 1, the 95% confidence intervals overlapped.

The second novel observation from our study was that sibling-to-sibling transmission may have been the primary mode of CMV acquisition among African-American children and adolescents. This was very different than we previously observed among Caucasian children in day care [12]. For those Caucasian children, child-to-child transmission of CMV acquired in day care was very common but sibling-to-sibling transmission at home seldom occurred. In the current study only 19 children reported day care attendance. Children not in day care probably spend more time at home with their siblings than occurs for children attending day care. Since child-to child transmission of CMV requires prolonged and frequent contact; the association of seroprevalence rates among African-American siblings, most of whom received home care, may represent sibling-to sibling transmission.

In our study 91% per cent of the caregivers were the biological mothers and over 70% were seropositive. Thus our data further indicate CMV acquisition by children from maternal sources such cervical-vaginal secretions, breast milk, or saliva is unlikely to account for the high rate of CMV infections among African- American adults. If infection from maternal sources occurred frequently, the children we observed should have had high CMV seroprevalence rates.

## Conclusion

We observed that the current generation of African-American children had CMV seroprevalence rates by age 20 years of less than one-half of that of their mothers. Our data suggest that sibling-to-sibling transmission was a possible source of CMV infections for the children. If our observations for Richmond VA represent what has occurred nationwide, a large proportion of the next generation of African-American women will be susceptible to a primary CMV infection during pregnancy and will benefit from a CMV vaccine. Exposure to household children is a major risk factor for congenital CMV infection [10]. Since we observed that 92% of infants are seronegative, infant immunization may be optimal.

## Competing interests

The authors declare that they have no competing interests.

## Authors' contributions

IRW, AMB and SPA all contributed to the design of the study and the data analysis. IRW performed subject enrollment, sample collection, and laboratory assays. All authors read and approved final manuscript.

## Pre-publication history

The pre-publication history for this paper can be accessed here:


